# Transitions in health insurance among continuously insured patients with schizophrenia

**DOI:** 10.1038/s41537-024-00446-4

**Published:** 2024-02-26

**Authors:** Brittany L. Ranchoff, Chanup Jeung, John E. Zeber, Gregory E. Simon, Keith M. Ericson, Jing Qian, Kimberley H. Geissler

**Affiliations:** 1 School of Public Health and Health Sciences, University of Massachusetts Amherst, Amherst, MA USA; 2 School of Public Health, State University of New York at Albany, Albany, NY USA; 3 Kaiser Permanente Washington Health Research Institute, Seattle, WA USA; 4 National Bureau for Economic Research, Cambridge, MA USA; 5 Boston University Questrom School of Business, Boston, MA USA; 6 Department of Healthcare Delivery and Population Sciences, UMass Chan Medical School-Baystate, Springfield, MA USA

**Keywords:** Schizophrenia, Diseases

## Abstract

Changes in health insurance coverage may disrupt access to and continuity of care, even for those who remain insured. Continuity of care is especially important in schizophrenia, which requires ongoing medical and pharmaceutical treatment. However, little is known about continuity of insurance coverage among those with schizophrenia. The objective was to examine the probability of insurance transitions for individuals with schizophrenia who were continuously insured and whether this varied across insurance types. The Massachusetts All-Payer Claims Database identified individuals with schizophrenia aged 18–64 who were continuously insured during a two-year period between 2014 and 2018. A logistic regression estimated the association of having an insurance transition – defined as having a change in insurance type – with insurance type at the start of the period, adjusting for age, sex, ZIP code in the lowest quartile of median income, and ZIP code with concentrated poverty. Overall, 15.1% had at least one insurance transition across a 24-month period. Insurance transitions were most frequent among those with plans from the Marketplace. In regression adjusted results, individuals covered by the traditional Medicaid program were 20.2 percentage points [pp] (95% confidence interval [CI]: 24.6 pp, 15.9 pp) less likely to have an insurance transition than those who were insured by a Marketplace plan. Insurance transitions among individuals with schizophrenia were common, with more than one in six people having at least one transition in insurance type during a two-year period. Given that even continuously insured individuals with schizophrenia commonly experience insurance transitions, attention to insurance transitions as a barrier to care access and continuity is warranted.

## Introduction

Access to care and continuity of care is important for people with schizophrenia^1^, which requires ongoing medical and pharmaceutical treatment. Schizophrenia is a chronic severe mental illness with an estimated prevalence between 0.25% and 0.64% in the United States (US)^2^. In the US, healthcare access is strongly connected to health insurance coverage^3^. Health insurance is a key component of timely access to care, timely diagnosis, and on-going treatment. Non-elderly adults with schizophrenia have high rates of public insurance coverage (e.g., Medicaid, Medicare, or dual Medicaid and Medicare)^4–6^, but also rely on private coverage. The ACA increased insurance coverage of individuals with schizophrenia with 96% of those diagnosed with schizophrenia having insurance coverage in the 2014–2020 period^5^, which is a much higher rate than the overall population^7^.

Insurance disruptions result when individuals experience a change in their health insurance coverage type or specific insurance plan or experience a period of uninsurance. All insurance disruptions can negatively impact access to care and continuity of care for individuals, even for those who remain insured. For example, switching insurance types or insurance plans may require changing healthcare providers due to changes in the included provider network^8,9^. Studies have found that individuals who experience insurance disruptions – either changes in insurance type or periods of uninsurance – have worse access to care and outcomes than those who are stably insured by the same insurance type^9–13^. Prior research has shown that switching insurance type and coverage gaps, especially longer gaps, are associated with more healthcare utilization that might indicate crisis or deterioration, such as inpatient visits and emergency room visits, including among individuals with schizophrenia or serious mental illness^13–19^. Additionally, longer enrollment with an insurer may create incentives for insurers to provide better preventive care that may not have immediate impacts but be cost-saving in the future; if enrollees are frequently changing insurance types or plans, these incentives for insurers are blunted^20,21^.

Prior research has found that individuals with serious mental illness experience frequent insurance disruptions^22–24^, and individuals with schizophrenia are more likely to experience an insurance disruption – often a coverage gap – compared to other types of serious mental illness^25^. However, most studies examining insurance stability for those with schizophrenia were conducted before the ACA, limited to those with either first episode psychosis or first diagnosis of schizophrenia, or limited to young adults^22–24^. In the most closely related study, Pesa and colleagues focused on a small sample of young adults (18-34 years) in Colorado with private insurance at the start of period and a new schizophrenia diagnosis, requiring one year of observation prior to observing a diagnosis. They found that more than half of this selected sample experienced changes in insurance type over a 48-month period^26^. Although studies have shown that low-income adults in general are less likely to experience periods of uninsurance after the ACA^27,28^, much less is known about insurance stability among those who remain continuously insured, including the broader population of non-elderly adults with schizophrenia. Adults with schizophrenia are disproportionately publicly insured and less likely to be uninsured than the general working-age adult population^5^; however, little is known about whether they experience transitions among different insurance types.

To better understand insurance dynamics for insured non-elderly adults with schizophrenia, we used the Massachusetts All-Payer Claims Database (APCD) to examine insurance transitions for adult individuals (18–64 years) with schizophrenia who were continuously insured over a two-year period.

## Methods

### Data

We used the Massachusetts APCD v8.0, obtained from the Massachusetts Center for Health Information and Analysis, from 2014 to 2018 for our analysis. The APCD contains health claims data and insurance eligibility details from public and many private insurance providers in Massachusetts^29,30^. There is full reporting by Traditional Medicaid and Medicaid managed care insurers, Marketplace plans, the Health Safety Net, and Medicare Advantage; there is partial reporting for private insurers as reporting is optional for self-insured employers. The Massachusetts Center for Health Information and Analysis uses a proprietary algorithm to match individuals across insurance carriers over time^31^; we conduct additional analyses to remove records that may represent incorrect matches. Eligibility records for those covered by Traditional Medicare are not included, with the exception of those enrolled in integrated Medicare-Medicaid coverage.

### Analytic sample

We identified adults with schizophrenia as those who had at least one claim with a principal or secondary diagnosis of schizophrenia based on *International Classification of Disease, 9th Revision (ICD-9)* and *ICD-10* diagnosis codes (ICD-9: 295.X excluding 295.7; ICD-10: F20.X)^32,33^. We limited to adults with schizophrenia who were 18 to 64 years old and a Massachusetts resident for the entire 2-year period. We further limited the sample to individuals who were continuously insured by one or more included plans during the two-year period. We made this decision as we cannot disentangle the reasons patients who transition to no longer being observed in the Massachusetts APCD; for example, individuals may become unobserved if they transition to a non-reporting private insurance plan or a Traditional Medicare plan, or they may become unobserved if they become uninsured. Individuals with data errors that could indicate issues with the unique identifier linkage process were excluded from the analysis.

### Measures

Our primary outcome of interest was a binary indicator of any transition in insurance type in each person-period. We examine transitions among seven categories of insurance coverage: (1) private; (2) Traditional Medicaid; (3) Medicaid managed care; (4) plans from the health insurance marketplace; (5) Health Safety Net; (6) Medicare Advantage; and (7) integrated Medicare and Medicaid. Each period included two consecutive calendar years, which we chose to observe a multi-year period allowing for analysis of within versus across-year transitions. In our study, we had four 24-month periods (2014–2015; 2015–2016; 2016–2017; 2017–2018), which means each patient could have been included for up to four periods. An insurance transition was defined as a change in insurance type between adjacent months in the 2-year period. For example, an insurance transition could include changing from private insurance to public insurance. If multiple transitions between types were observed, we counted these even if the individual was transitioning back to the insurance type in which they were enrolled at the beginning of the period. Due to data limitations, we did not consider changes between health plans within a specific insurance type to be an insurance transition.

Massachusetts Medicaid beneficiaries generally have the choice to select either Traditional Medicaid administered by the state or a plan from a Medicaid-managed care organization. While individuals who chose the traditional state-administered program can switch into a Medicaid-managed care plan at any time, individuals who are enrolled in a Medicaid-managed care plan can generally only change during the annual plan enrollment periods^34,35^. Medicaid redetermination and moving between regions in the state also allows beneficiaries to switch between Medicaid plan types^36^.

Marketplace plans are those plans purchased from the health insurance exchange or marketplace created by the ACA. The Health Safety Net is a Massachusetts program covering specific health services at acute care hospitals or community health centers for individuals who are uninsured and underinsured; however, it does not qualify as minimum essential coverage under ACA requirements^37^. Individuals enrolled in the Health Safety Net were included as they had access to some care and can be observed in the data; however, in other states, these individuals are likely to be uninsured. The dataset includes Medicare Advantage, but does not include eligibility or claims information for those insured by Traditional Medicare. In October 2013, MassHealth (Medicaid) and Medicare created One Care, which is Medicare plus MassHealth for dual-eligible individuals who are between 21 to 64 years old^38,39^. Based on enrollment reports, 22.8% who are eligible enroll in One Care^40^; patients with One Care or Medicare Advantage plus secondary Medicaid would be observed in the dataset while individuals with Traditional Medicare plus secondary Medicaid outside of One Care would not be observed.

In addition, we created a binary measure for patient sex and a categorical measure for age at the start of the 2-year period. To understand the association of insurance transitions with socioeconomic status, we used patient 5-digit ZIP code to identify those living in areas in the lowest quartile of median household income^41^ and areas with concentrated poverty^42^. Concentrated poverty was defined as a ZIP code with an overall poverty rate greater than 30%^43^.

### Statistical analysis

We calculated descriptive statistics to compare any insurance transition by sample characteristics and used chi-square tests to evaluate the significance of differences between those with and without an insurance transition. Next, we estimated a multivariable logistic regression model for the binary outcome of any insurance transition with the primary independent variable being a categorical measure of insurance type at the start of the 2-year period. We included covariates of age, sex, residence in a ZIP code in the lowest quartile of median income, and residence in a ZIP code with concentrated poverty. We presented predicted probabilities of any insurance transition. Lastly, we displayed any insurance transition by insurance type using a Sankey diagram. To further display information about enrollment duration, we analyzed the timing of the initial transition for those with at least one transition, and constructed a figure showing enrollment durations in each insurance type.

We conducted three sensitivity analyses. We first repeated the main analysis with the sample but combined the Medicaid coverage into one category rather than separating into Medicaid and Medicaid managed care as in the primary analysis. Next, we repeat the analysis removing the 2014 to 2015 period to see how the results changed for dual-eligible enrollees given that One Care began in October 2013 and was still coming into effect in 2014. Lastly, we repeat the analysis using two-year periods that do not align with calendar years to determine whether our results are not sensitive to the start month of the analysis. We defined a person-period as a period of two consecutive calendar years starting on April 1 of a given year.

Standard errors were clustered at the 5-digit patient ZIP code. Standard errors for predicted probabilities are calculated using the delta method. An alpha of 0.05 was defined as statistically significant. All analysis was conducted in SAS Version 9.4 (Cary, NC) and Stata-MP 16.0 (College Station, TX). Our study was approved by the University of Massachusetts Amherst Institutional Review Board.

## Results

The analytic sample included 36,754 person-period observations for 19,217 unique individuals aged 18 to 64 in Massachusetts with a schizophrenia diagnosis (Supplementary Fig. 1). At the start of the study period, 39.0% were covered by Traditional Medicaid and 37.0% by Medicaid managed care (Table 1). Almost half (46.3%) of the sample resided in a ZIP code with the lowest-quartile median income, and 10.9% of patients lived in a ZIP code with concentrated poverty. Overall, more than one-sixth (15.1%) experienced at least one insurance transition over a 2-year period. Among those with any insurance transition, 70.9% experienced one insurance transition, 22.0% had two insurance transitions, and 7.1% had three or more insurance transitions. Insurance transitions were more frequent among those covered by Traditional Medicaid, Marketplace plans, and the Health Safety Net.

**Table 1:** Sample Characteristics (*N* = 36,754).

Characteristic	Overall (*N* = 36,754)		Insurance Transition in 2-year period				
			No (*n* = 31,218)		Yes (*n* = 5536)		*P* value
	*N*	%	*n*	%	*n*	%	
Outcomes							
Any transition			31218	84.9	5536	15.1	
Number of transitions							
None			31218	100	–	–	
1 transition			–	–	3924	70.9	
2 transitions			–	–	1218	22.0	
3 or more transitions			–	–	394	7.1	
Age, y							<0.001
18–25	5859	15.9	4891	15.7	968	17.5	
26–40	11403	31.0	9416	30.2	1987	35.9	
41–55	12291	33.4	10597	34.0	1694	30.6	
56–64	7201	19.6	6314	20.2	887	16.0	
Sex							0.545
Female	14455	39.3	12298	39.4	2157	39.0	
Male	22299	60.7	18920	60.6	3379	61.0	
Period (2-year period)							<0.001
2014–2015	9943	27.1	8522	27.3	1421	25.7	
2015–2016	10423	28.4	9108	29.2	1315	23.8	
2016–2017	7843	21.3	6962	22.3	881	15.9	
2017–2018	8545	23.3	6626	21.2	1919	34.7	
Health insurance type at start of period							<0.001
Private	5516	15.0	4975	15.9	541	9.8	
Traditional Medicaid	14318	39.0	11396	36.0	2922	52.8	
Medicaid managed care	13600	37.0	12438	39.8	1162	21.0	
Marketplace	854	2.3	491	1.6	363	6.6	
Health Safety Net	1329	3.6	880	2.8	449	8.1	
Medicare Advantage	633	1.7	605	1.9	28	0.5	
Integrated Medicare & Medicaid	504	1.4	433	1.4	71	1.3	
Residence in zip code with lowest-quartile median income							0.202
Yes	17025	46.3	14417	46.2	2608	47.1	
No	19729	53.7	16801	53.8	2928	52.9	
Residence in zip code with concentrated poverty							0.087
Yes	4013	10.9	3372	10.8	641	11.6	
No	32741	89.1	27846	89.2	4895	88.4	

**Fig. 1:**
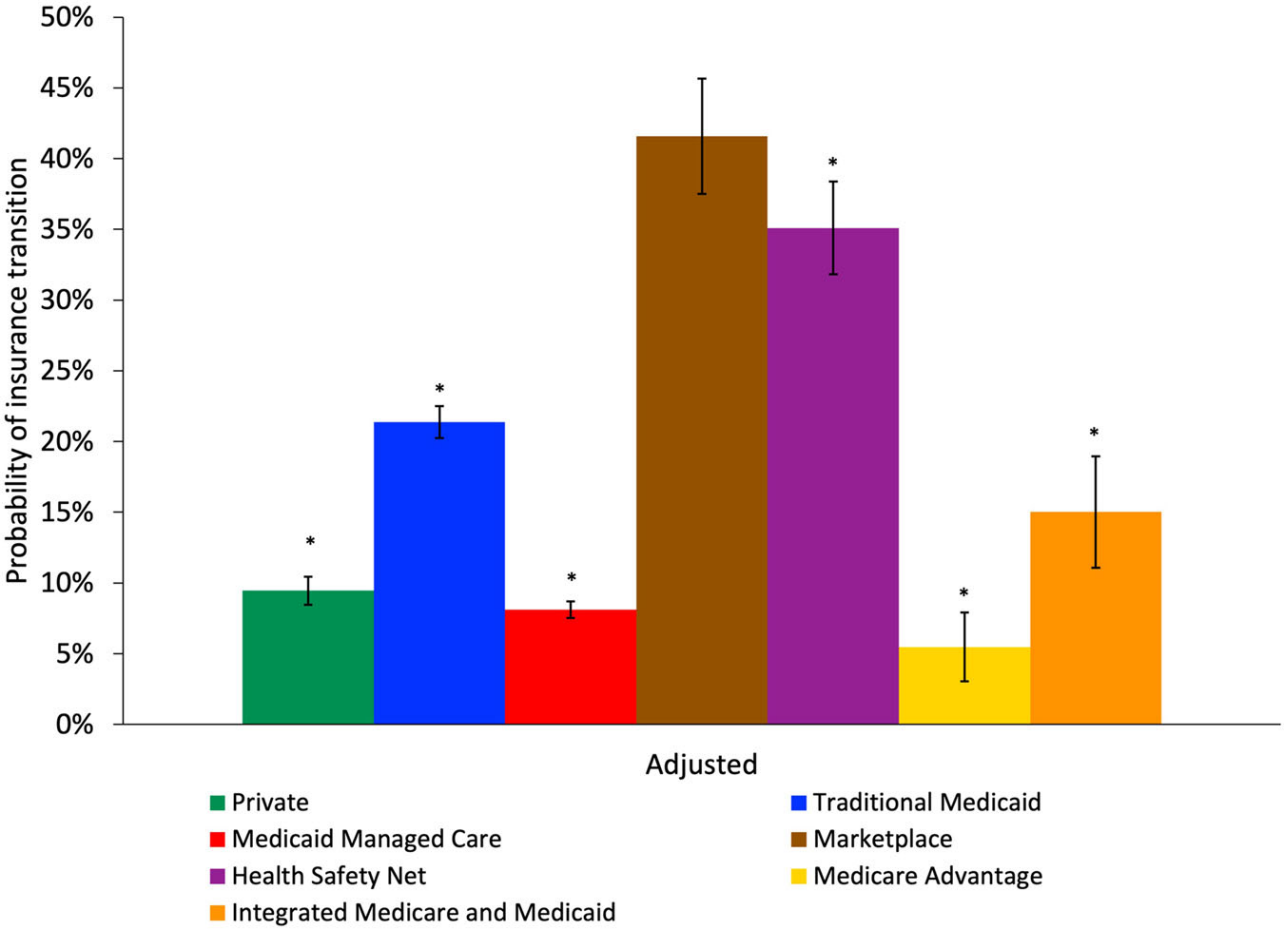
Adjusted rates of health insurance transition for individuals with schizophrenia by health insurance type at start of period. *N* = 36,754 person-period observations. Adjusted results control for age, sex, residence in a ZIP code in the lowest quartile of median income, and residence in a ZIP code with concentrated poverty. Standard errors are clustered at the 5-digit ZIP code level. *Indicates a statistically significant difference in predicted probability from the reference group (Marketplace insurance) at the 5% level. 95% Confidence intervals for each group are shown with vertical bars.

**Fig. 2:**
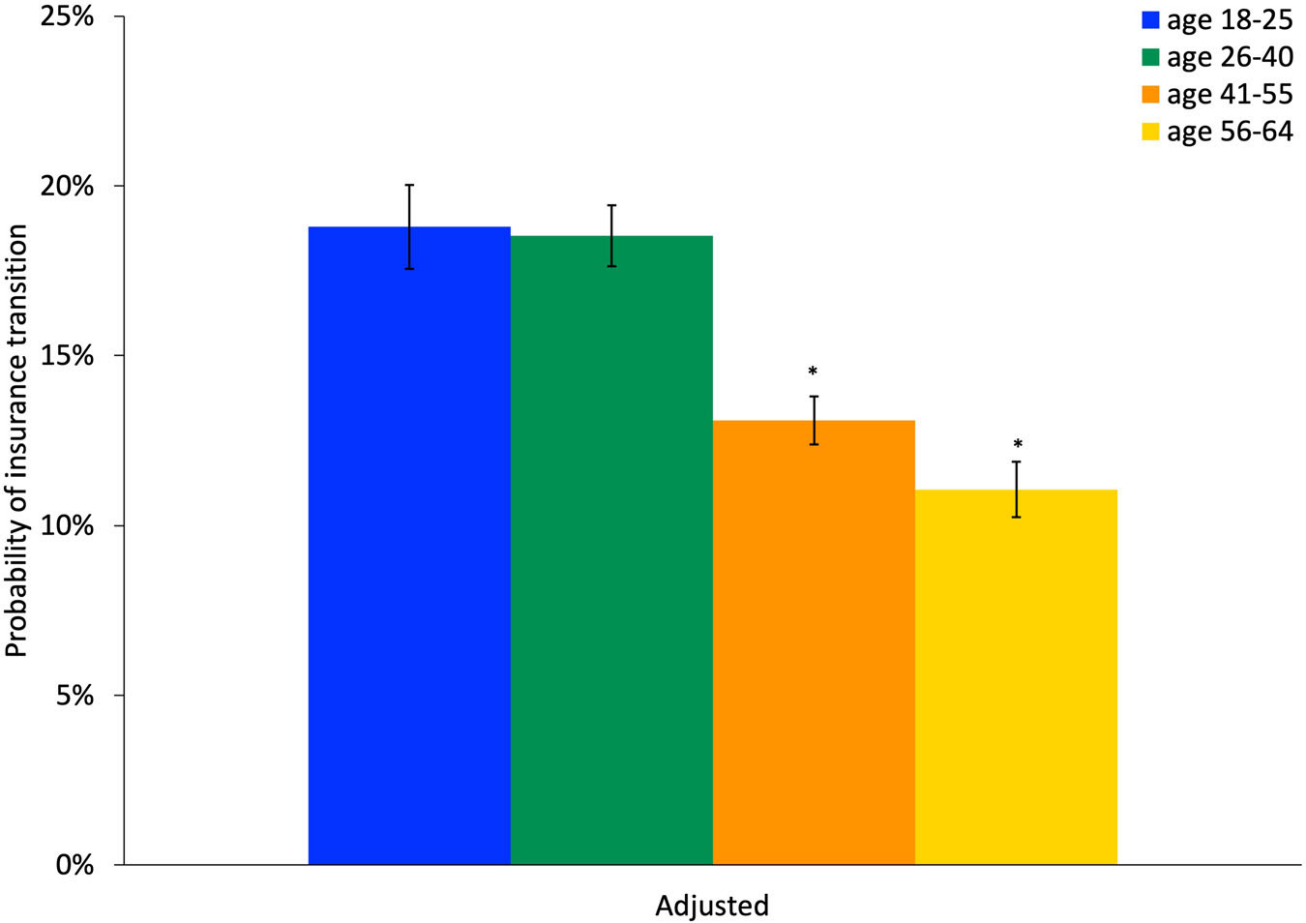
Adjusted rates of health insurance transition for individuals with schizophrenia by age. *N* = 36,754 person-period observations. Adjusted results control for insurance type at the start of the period, sex, residence in a ZIP code in the lowest quartile of median income, and residence in a ZIP code with concentrated poverty. Standard errors are clustered at the 5-digit ZIP code level. *Indicates a statistically significant difference in predicted probability from the reference group (age 18–25 years) at the 5% level. 95% Confidence intervals for each group are shown with vertical bars.

Figure 1 shows the regression-adjusted predicted probabilities of insurance transitions by insurance type at the start of period. In regression-adjusted results, insurance transitions were significantly less common among private insurance, Traditional Medicaid, Medicaid managed care, Health Safety Net, Medicare Advantage, and integrated Medicare and Medicaid coverage compared to individuals with Marketplace insurance (Fig. 1; full multivariate logistic regression results in Supplementary Table 1). The findings were similar in unadjusted analyses (Supplementary Fig. 2). In regression-adjusted results, individuals insured with Traditional Medicaid were 20.2 percentage points [pp] (95% Confidence Interval [CI]: −24.6 pp, −15.9 pp) less likely to have an insurance transition than those who were insured by a Marketplace plan (21.4% vs. 41.6%). In comparison, individuals insured with private insurance and Medicaid managed care plan were 32.1 pp (95% CI: −36.3 pp, −28.0 pp) and 33.5 pp (95% CI: −37.6 pp, −29.4 pp), respectively less likely to have an insurance transition compared to those who had Marketplace coverage (predicted probabilities of 9.5% private vs. 8.1% Medicaid managed care vs. 41.6% Marketplace). Insurance transitions were less common for older individuals in regression-adjusted results (Fig. 2), which was similar in unadjusted analyses (Supplementary Fig. 3). The regression-adjusted probability of a 56- to 64-year-old with schizophrenia having an insurance transition was 7.7 pp lower (95% CI: −9.1 pp, −6.4 pp) than an 18- to 25-year-old with schizophrenia.

**Fig. 3:**
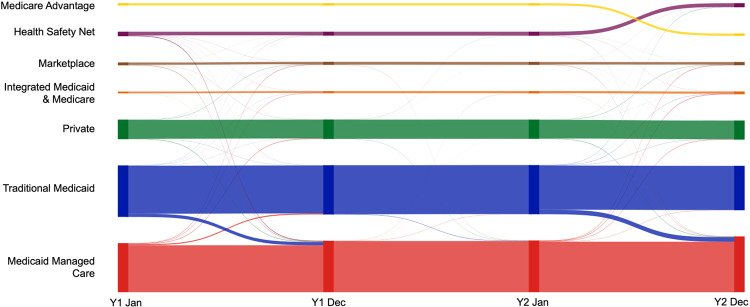
Health insurance transitions over the study period. For display purposes, all combinations with fewer than 11 observations were dropped (*N* = 467; 1.3%). Among those with Marketplace and Health Safety Net insurance at month 1, 7.7% and 11.4% were dropped, respectively.

Figure 3 displays the insurance transitions over the study period at four time points (Year 1 January, Year 1 December, Year 2 January, and Year 2 December). In the figure, it shows that insurance transitions happen not only between calendar years, but within calendar years as well. Supplementary Table 2a and b present transition matrices. The proportion of individuals who experienced an insurance transition increased over the period from 1.3% in Year 1 February to 15.1% in Year 2 December (Supplementary Fig. 4), with a modal duration of 14 months for those with at least one insurance transition (Supplementary Fig. 5).

**Fig. 4:**
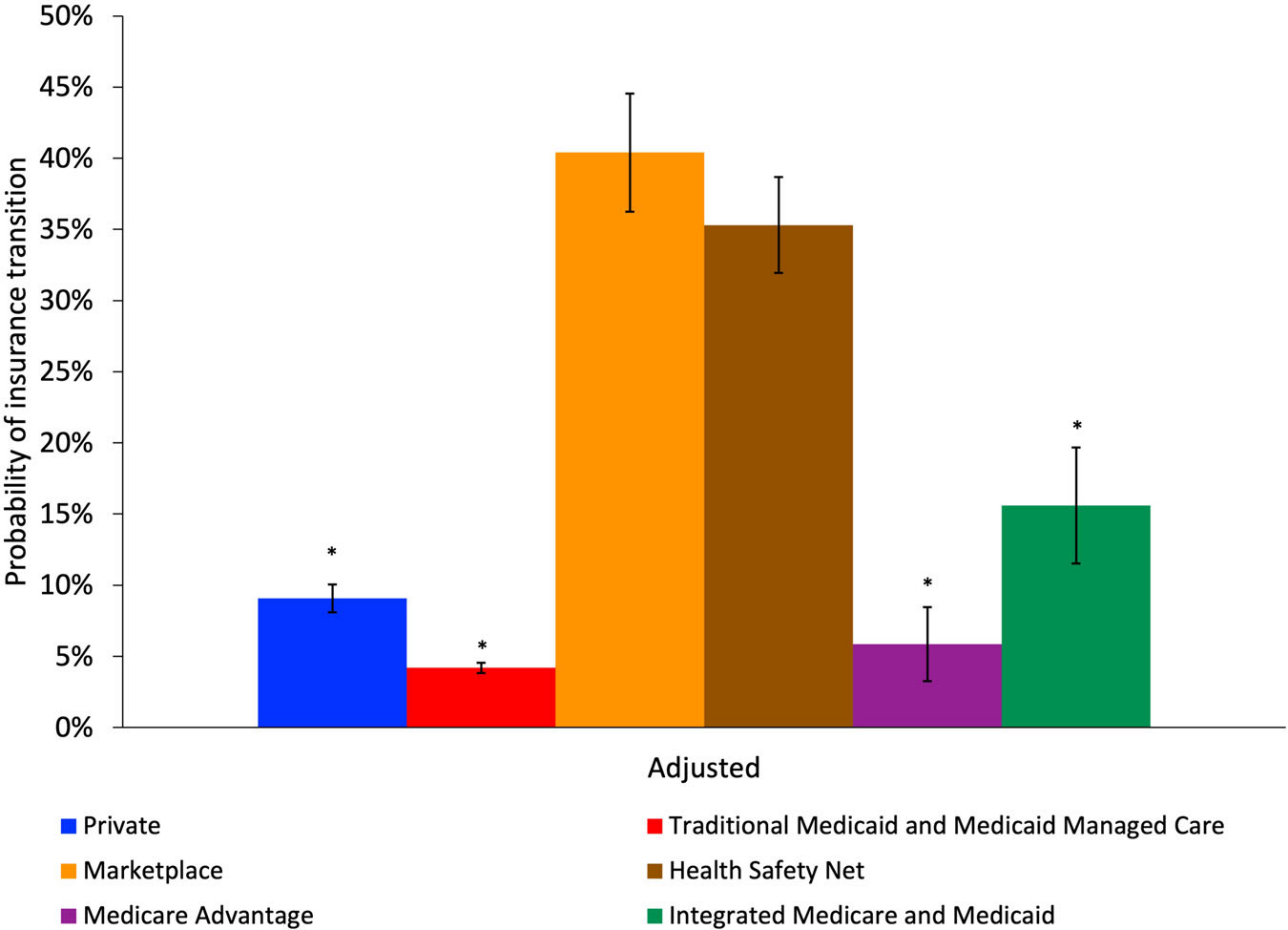
Adjusted rates of health insurance transition for sensitivity analysis combining Traditional Medicaid and Medicaid Managed Care for individuals with schizophrenia by health insurance type at start of period. *N* = 36,754 person-period observations. Adjusted results control for age, sex, residence in a ZIP code in the lowest quartile of median income, and residence in a ZIP code with concentrated poverty. Standard errors are clustered at the 5-digit ZIP code level. *Indicates a statistically significant difference in predicted probability from the reference group (Marketplace insurance) at the 5% level. 95% Confidence intervals for each group are shown with vertical bars.

In the sensitivity analysis combining Traditional Medicaid and Medicaid managed care, we found similar regression adjusted predicted probabilities of insurance transitions except for people insured with either Traditional Medicaid or Medicaid managed care (Fig. 4; full multivariate logistic regression results in Supplementary Table 3 and unadjusted results in Supplementary Fig. 6). In regression adjusted results, individuals insured with either Traditional Medicaid or Medicaid managed care were 36.2 pp (95% CI: −40.4 to −32.0 pp) less likely to have an insurance transition than those who were insured by a Marketplace plan (4.2% for Traditional Medicaid or Medicaid managed care vs. 40.4% Marketplace). We also display the insurance transitions over the study period for the sensitivity analysis combining Traditional Medicaid and Medicaid managed care (Supplementary Fig. 7). Similar to the main results, most insurance transitions do not happen between calendar years but occur during the calendar year.

In the sensitivity analysis removing the 2014 to 2015 period, the findings were consistent with the main analysis (Supplementary Table 4). In the adjusted regression results, individuals insured with integrated Medicare and Medicaid coverage were 19.6 pp (95% CI: −25.9 pp, −13.3 pp) less likely to have an insurance transition than those who were insured by a Marketplace plan.

In the sensitivity analysis changing the period to an April start, the findings were consistent with the main analysis (Supplementary Fig. 8). Overall, 15.8% experienced at least one insurance transition over a 2-year period, and regression adjusted results were similar.

## Discussion

Overall, we found one in six continuously insured people with schizophrenia had at least one insurance transition among insurance types during a two-year period. Our results indicate that insurance transitions were particularly common for individuals with Marketplace plans, those covered by the Traditional Medicaid program, and those in the Health Safety Net. Among those with at least one insurance transition, we found approximately 30% had more than one insurance transition over a 2-year period.

Our findings align with other studies examining individuals with serious mental illness experiencing frequent insurance disruptions, including either moving between insurance types or experiencing coverage gaps^22–24^. We found that insurance transitions between insurance types among non-elderly continuously insured adults with schizophrenia are frequent, although less common than in the most closely related study, which was done in a more limited sample. In that study, Pesa and colleagues found 54 and 85% of young adults with a newly observed schizophrenia diagnosis experienced a change in insurance type after 12 months and 48 months, respectively^26^. Given their focus on young adults with private insurance at start of period – something that is relatively uncommon among those with schizophrenia^5^, they found the most common insurance transition was from private insurance plan to a Medicaid plan^26^, which might be impacted by the ACA provision of dependent coverage ending at age 26 or new qualification for public insurance programs after a schizophrenia diagnosis. We similarly found that younger individuals were more likely to experience an insurance transition compared to oldest age category (56–64) with schizophrenia.

Next, insurance transitions were particularly common among those covered by the Traditional Medicaid program while people with a Medicaid managed care plan had more stable coverage. Our findings are consistent with the literature indicating that people covered by Medicaid plans are particularly vulnerable to insurance disruptions – either a change in insurance type or a coverage gap – including people who are dually enrolled in Medicaid and Medicare^44–48^. The literature has also shown that Traditional Medicaid is more vulnerable to insurance disruptions compared to Medicaid-managed care^45^. Medicaid insurance disruptions – primarily coverage gaps – are associated with increased care utilization, including inpatient hospitalization and outpatient services^14–18^. Our findings show that after the ACA, continuously insured individuals with schizophrenia covered by Medicaid experience high rates of insurance transitions; thus, examining Medicaid eligibility and redetermination policies might be beneficial to understand why these transitions occur^49^. For instance, automatic re-enrollment policies have been seen as beneficial tools in preventing insurance disruptions^50^; although primarily designed to avoid coverage gaps, these automatic re-enrollment policies may have a role in reducing insurance transitions as well.

Besides insurance transitions being common among those with Traditional Medicaid, transitions were also frequent for individuals with a Marketplace plan. This finding is consistent with prior research showing Marketplace plans have high rates of disruptions^9,51^, which can occur after relatively small changes in income or changes in family status. Increasing the stability of Marketplace coverage may be a policy priority given high-risk populations insured by this coverage.

Overall, individuals with schizophrenia require consistent treatment to address symptoms and decrease untreated psychosis^52^. Increased provider continuity for mental health care has been associated with lower costs for Medicaid plans^53^. Health insurance transitions, even without gaps in coverage, may result in negative impacts in care utilization and outcomes^9–13^. In addition, insurance type may impact the type and amount of care received and whether continuity of care can be maintained with their clinicians^54,55^. Given provider networks differ across plans and insurance type^8,9,56,57^, continuity of care, especially for more specialized care, might be difficult for those who experience an insurance transition despite being continuously insured.

### Limitations

Our study has several limitations. First, our study was limited to Massachusetts and thus may not be representative of the population with schizophrenia nationally. However, although Massachusetts has the lowest uninsurance rate in the US^7^, uninsurance rates among those with schizophrenia nationally are very low after the ACA^5^. Given the limitation of our findings to Massachusetts, there may be different types of insurance transitions in other states based on the setup and eligibility requirements of their state Medicaid program and programs for dual eligible, higher rates of Medicaid disruptions, and/or have a smaller proportion of the population with schizophrenia who is continuously insured. Second, our data do not allow us to discern reasons for insurance transitions, so identifying policy responses to ensure more coverage stability is difficult. However, attention by state Medicaid programs to minimizing disruptions among different Medicaid types may be a key priority to maintain continuity. Third, some individuals have multiple eligibility records for the same month in the Massachusetts APCD. We have used a prioritization algorithm to ensure that we maintain continuity of insurance type, when possible, but multiple eligibility records may be an indicator of not disenrolling from a plan from which one is no longer actively receiving benefits. For example, an individual could still be enrolled in Medicaid but potentially move, die, or be incarcerated. One study found that only 3% of individuals with serious mental illness who were incarcerated were disenrolled from their Medicaid plan^58^. Thus, this Medicaid eligibility record might persist for some time – other insurance types may be quicker to remove eligibility, and thus we may overestimate transitions back into Medicaid coverage. Fourth, our study does not examine changes between plans within an insurance type. While an individual could have the same insurance type throughout the period, they could have experienced insurance transition by switching health plans. Switching health plans within an insurance type could also impact access to care and continuity of care due to differences in provider network and benefits^59^, and thus our estimates are a conservative measure of insurance transitions. Fifth, due to *Gobeille v Liberty Mutual Insurance Co*., the APCD does not contain full reporting by self-insured employers^60^; additionally, due to access restrictions, it does not include those with Traditional Medicare. As a result, we do not observe individuals with continuous insurance from non-reporting insurance plans, which may result in overstating or understating the insurance transitions for people with schizophrenia. However, given most individuals with schizophrenia are enrolled in public insurance, the lack of private non-reporting insurance plans is less of a concern; among those otherwise eligible who begin the two-year period in private insurance, 7% of those dropped from the dataset altogether over the period (author’s analysis).

## Conclusions

Continuously insured adults with schizophrenia encounter frequent insurance transitions over a two-year period, which may leave them vulnerable to barriers in accessing care and high-quality care. These transitions were particularly common among those covered by the Traditional Medicaid program and by Marketplace plans, suggesting that attention to maintaining stable insurance within the Traditional Medicaid program and the Marketplace may be important to improve care for this population. The insurance transitions may lead to decreased care continuity and access to care for this population. Further research is needed to identify the impacts of insurance transitions on care utilization among individuals with schizophrenia.

### Supplementary information

Supplementary Information

## Data Availability

The Massachusetts All-Payer Claims Database is available for purchase to qualified researchers from the Massachusetts Center for Health Information and Analysis.

## Code availability

The codes used for this study can be accessed upon reasonable request to the corresponding author.
