# Supplementary material for: Transitions in health insurance among continuously insured patients with schizophrenia
Source: Schizophrenia (Heidelb). 2024 Feb 26;10(1):25. doi: 10.1038/s41537-024-00446-4 (PMC10897200; doi:10.1038/s41537-024-00446-4)
Supplement: Supplementary file 1 — Supplementary Information [file 41537_2024_446_MOESM1_ESM.docx]

**Supplementary Information**

**Supplementary Figure 1. Study Flow Diagram**

**Supplementary Table 1. Full Regression Results and Average Marginal Effect**

| **Outcome:** Any Transition | **aOR** | **Standard Error** | **95% CI** | | ***P* value** | **Average Marginal Effect** | **Standard Error** | **95% CI** | | ***P* value** |
| --- | --- | --- | --- | --- | --- | --- | --- | --- | --- | --- |
|  |  |  | **LCI** | **UCI** |  |  |  | **LCI** | **UCI** |  |
| **Health insurance type at start of period (ref = Marketplace)** |  |  |  |  |  |  |  |  |  |  |
| Private | 0.14 | 0.01 | 0.12 | 0.18 | <.001 | -0.32 | 0.02 | -0.36 | -0.28 | <.001 |
| Traditional Medicaid | 0.38 | 0.04 | 0.31 | 0.45 | <.001 | -0.20 | 0.02 | -0.25 | -0.16 | <.001 |
| Medicaid managed care | 0.12 | 0.01 | 0.10 | 0.15 | <.001 | -0.33 | 0.02 | -0.38 | -0.29 | <.001 |
| Health Safety Net | 0.76 | 0.08 | 0.61 | 0.94 | .012 | -0.06 | 0.03 | -0.12 | -0.01 | .013 |
| Medicare Advantage | 0.08 | 0.02 | 0.05 | 0.13 | <.001 | -0.36 | 0.02 | -0.41 | -0.31 | <.001 |
| Integrated Medicare & Medicaid | 0.24 | 0.04 | 0.17 | 0.35 | <.001 | -0.27 | 0.03 | -0.32 | -0.21 | <.001 |
| **Age, y (ref = 18-25)** |  |  |  |  |  |  |  |  |  |  |
| 26-40 | 0.98 | 0.05 | 0.89 | 1.09 | .724 | -0.003 | 0.01 | -0.02 | 0.01 | .724 |
| 41-55 | 0.63 | 0.03 | 0.57 | 0.70 | <.001 | -0.06 | 0.01 | -0.07 | -0.04 | <.001 |
| 56-64 | 0.52 | 0.03 | 0.46 | 0.58 | <.001 | -0.08 | 0.01 | -0.09 | -0.06 | <.001 |
| **Female sex (ref = male sex)** | 1.05 | 0.03 | 0.98 | 1.12 | .164 | 0.01 | 0.00 | -0.002 | 0.01 | .165 |
| **Residence in zip code with lowest-quartile median income (ref = No)** | 0.96 | 0.05 | 0.87 | 1.06 | .416 | 0.004 | 0.01 | -0.02 | 0.01 | .145 |
| **Residence in zip code with concentrated poverty (ref = No)** | 1.08 | 0.11 | 0.88 | 1.32 | .461 | 0.0094 | 0.01 | -0.02 | 0.03 | .469 |

**Abbreviations:** aOR, adjusted odds ratio; CI, confidence interval; LCI, lower confidence interval; UCI, upper confidence interval

**Supplementary Figure 2. Unadjusted Rates of Health Insurance Transition for Individuals with Schizophrenia by Health Insurance Type at Start of Period**


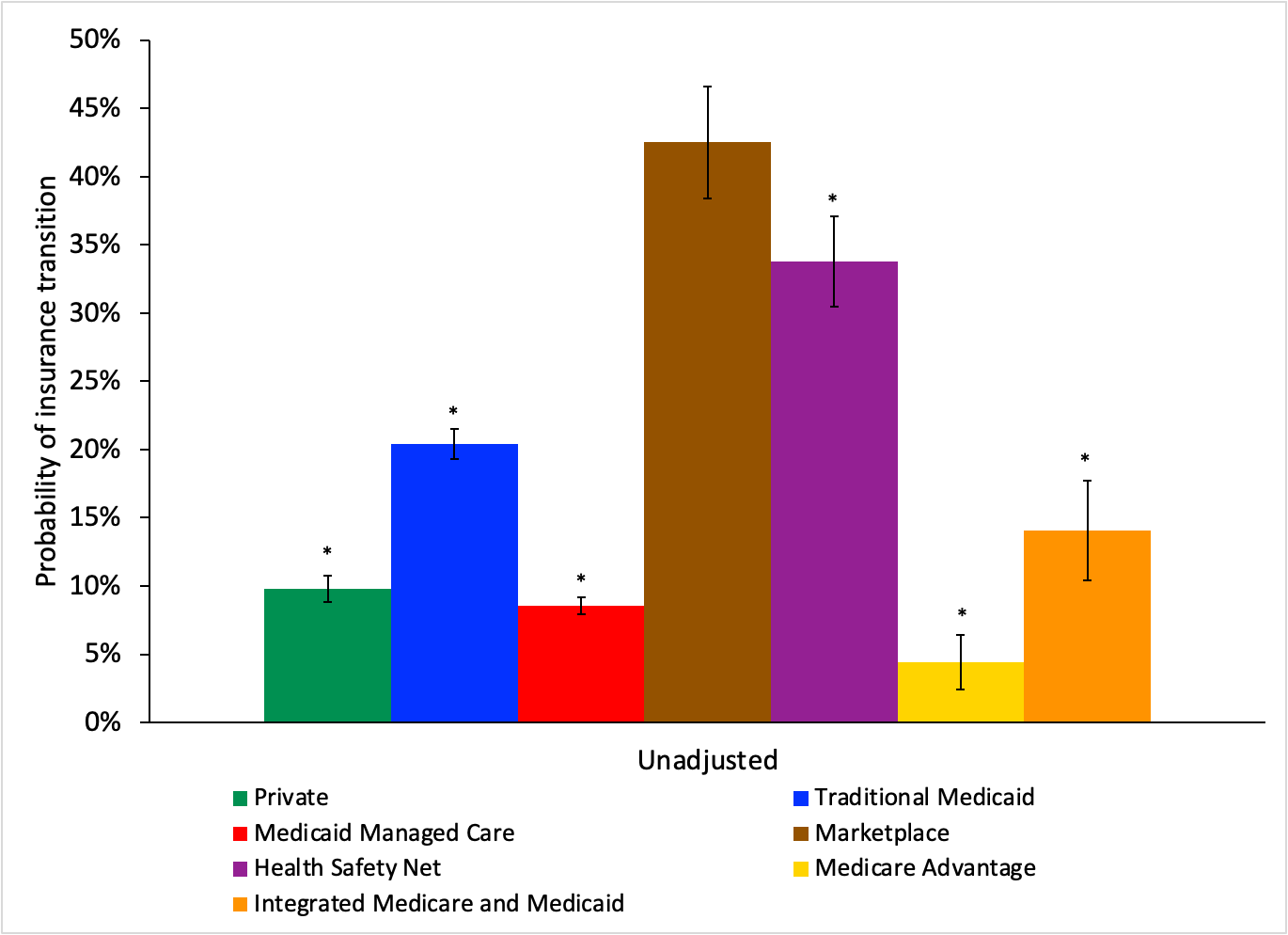


**Note:** N = 36,754 person-period observations. Standard errors are clustered at the 5-digit ZIP code level. *Indicates a statistically significant difference in predicted probability from the reference group (Marketplace insurance) at the 5% level. 95% Confidence intervals for each group are shown with vertical bars.

**Supplementary Figure 3. Unadjusted Rates of Health Insurance Transition for Individuals with Schizophrenia by Age**


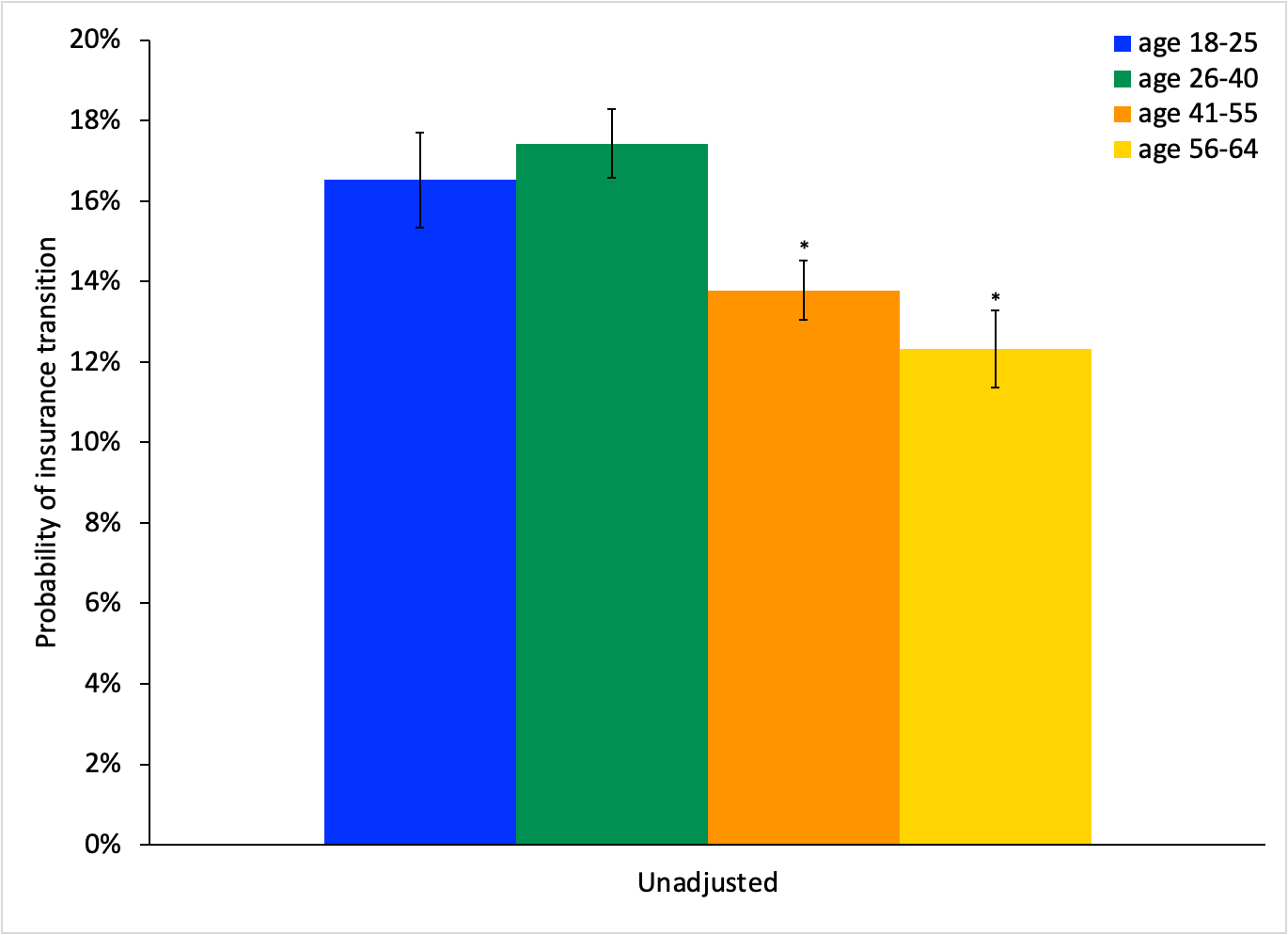


**Note:** N = 36,754 person-period observations. Standard errors are clustered at the 5-digit ZIP code level. *Indicates a statistically significant difference in predicted probability from the reference group (age 18-25 years) at the 5% level. 95% Confidence intervals for each group are shown with vertical bars.

**Supplementary Table 2. Supplemental Information for Sankey Diagram**

**Supplementary Table 2a. Baseline Health Insurance Type (Year 1 January) by Health Insurance Type (Year 2 December)**

| **Health Insurance Type at Year 1 January** | **Health Insurance Type at Year 2 December** | | | | | | | **Total of Displayed Cells** |
| --- | --- | --- | --- | --- | --- | --- | --- | --- |
|  | Private | Traditional Medicaid | Medicaid managed care | Marketplace | Health Safety Net | Medicare Advantage | Integrated Medicare & Medicaid |  |
| Private | 5044 | 37 | 190 | 137 | 70 | 16 | 22 | **5516** |
| Traditional Medicaid | 57 | 11940 | 2126 | 46 | 75 | * | 73 | **14317** |
| Medicaid managed care | 152 | 279 | 12751 | 164 | 23 | 22 | 209 | **13600** |
| Marketplace | 56 | 30 | 169 | 542 | 52 | * | * | **849** |
| Health Safety Net | 28 | 32 | 207 | 46 | 952 | 16 | 48 | **1329** |
| Medicare Advantage | * | * | * | * | 16 | 609 | * | **625** |
| Integrated Medicare & Medicaid | * | * | 54 | * | * | * | 449 | **503** |
| **Total of Displayed Cells** | **5337** | **12318** | **15497** | **935** | **1188** | **663** | **801** | **36739** |

**Supplementary Table 2b. Baseline Health Insurance Type (Year 1 January) by Health Insurance Type (Year 1 December)**

| **Health Insurance Type at Year 1 January** | **Health Insurance Type at Year 1 December** | | | | | | | **Total of Displayed Cells** |
| --- | --- | --- | --- | --- | --- | --- | --- | --- |
|  | Private | Traditional Medicaid | Medicaid managed care | Marketplace | Health Safety Net | Medicare Advantage | Integrated Medicare & Medicaid |  |
| Private | 5260 | 45 | 97 | 67 | 36 | * | * | **5505** |
| Traditional Medicaid | 38 | 13282 | 899 | 25 | 52 | * | 22 | **14318** |
| Medicaid managed care | 107 | 293 | 12997 | 101 | 18 | * | 76 | **13592** |
| Marketplace | 38 | 15 | 104 | 664 | 31 | * | * | **852** |
| Health Safety Net | 20 | 30 | 168 | 42 | 1037 | 11 | 21 | **1329** |
| Medicare Advantage | * | * | * | * | * | 620 | * | **620** |
| Integrated Medicare & Medicaid | * | * | 31 | * | * | * | 471 | **502** |
| **Total of Displayed Cells** | **5463** | **13666** | **14296** | **899** | **1174** | **631** | **590** | **36718** |

**Note:** We have redacted cells to preserve patient confidentiality. Cells that are calculated based on fewer than 11 individuals have been redacted, as indicated by a * symbol. The total reflects the “Total of Displayed Cells,” meaning the total is the sum of the unredacted cells.

**Supplementary Figure 4. Timing of First Insurance Transition from Health Insurance at Start of Period**


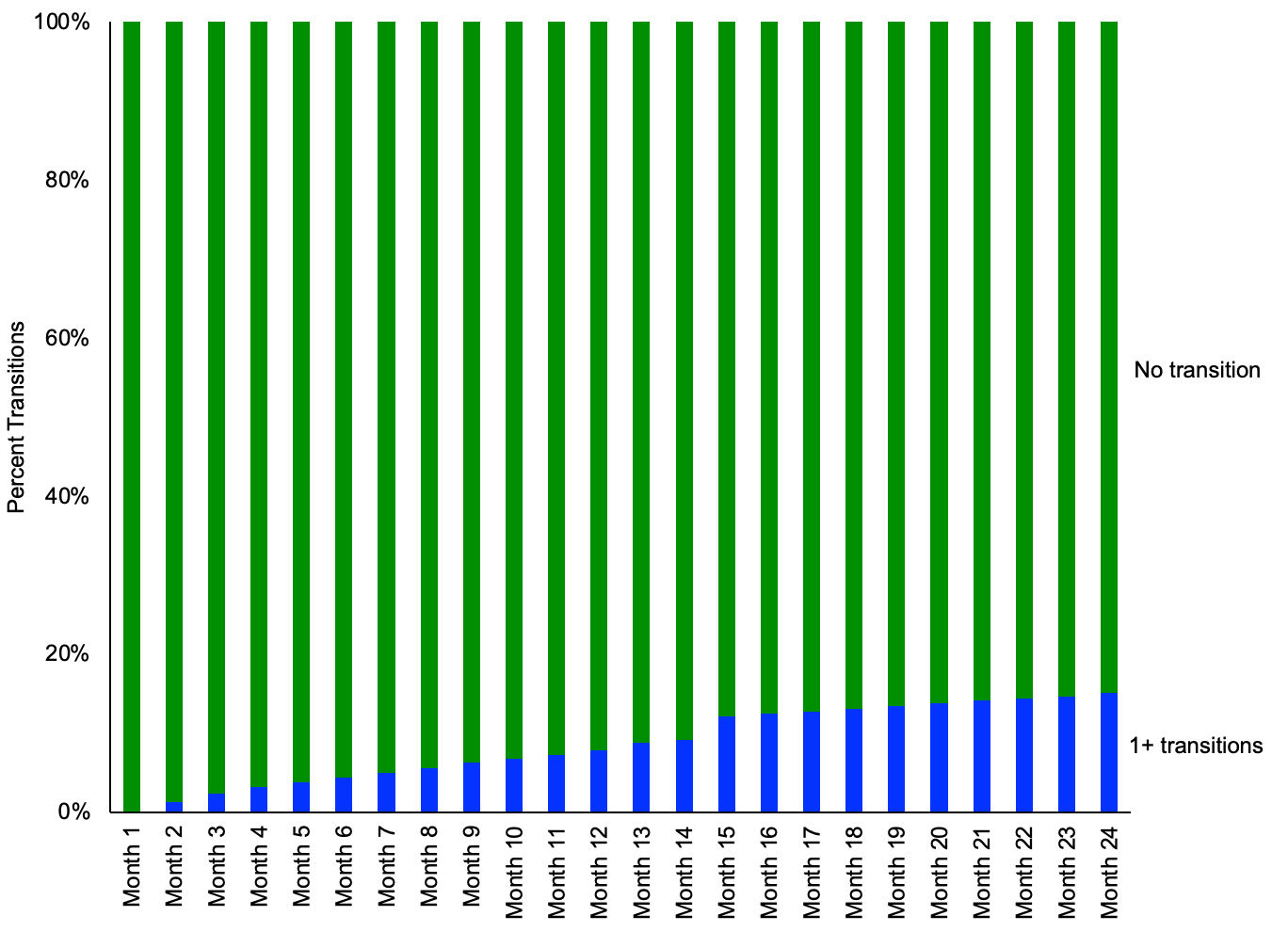


**Note:** Month 1 is Year 1 January and Month 24 is Year 2 December.

**Supplementary Figure 5. Number of Months in an Insurance type among Individuals with at least one Insurance Transition**


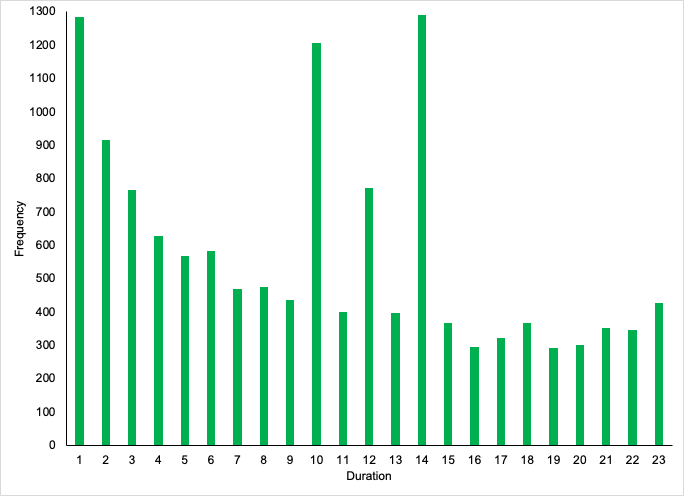


**Note:** Duration was calculated to show the enrollment length in each insurance type. If an individual had 2 transitions, they would have a total of 3 durations in the figure. The sum of enrollment durations for each individual is equal to 24 months. If an individual transitions from one type to another and back to the original type, we include all three enrollment durations in the figure rather than combining the durations in the same type.

**Supplementary Table 3. Full Regression Results for Sensitivity Analysis Combining Traditional Medicaid and Medicaid Managed Care**

| **Outcome:** Any Transition | **aOR** | **Standard Error** | **95% CI** | | ***P* value** | **Average Marginal Effect** | **Standard Error** | **95% CI** | | ***P* value** |
| --- | --- | --- | --- | --- | --- | --- | --- | --- | --- | --- |
|  |  |  | **LCI** | **UCI** |  |  |  | **LCI** | **UCI** |  |
| **Health insurance type at start of period (ref = Marketplace)** |  |  |  |  |  |  |  |  |  |  |
| Private | 0.14 | 0.02 | 0.11 | 0.17 | <.001 | -0.31 | 0.02 | -0.36 | -0.27 | <.001 |
| Traditional Medicaid & Medicaid managed care | 0.06 | 0.01 | 0.05 | 0.07 | <.001 | -0.36 | 0.02 | -0.40 | -0.32 | <.001 |
| Health Safety Net | 0.80 | 0.09 | 0.64 | 1.00 | .052 | -0.05 | 0.03 | -0.10 | 0.001 | .053 |
| Medicare Advantage | 0.09 | 0.02 | 0.05 | 0.15 | <.001 | -0.35 | 0.03 | -0.40 | -0.295 | <.001 |
| Integrated Medicare & Medicaid | 0.26 | 0.05 | 0.18 | 0.38 | <.001 | -0.25 | 0.03 | -0.31 | -0.19 | <.001 |
| **Age, y (ref = 18-25)** |  |  |  |  |  |  |  |  |  |  |
| 26-40 | 1.05 | 0.08 | 0.91 | 1.22 | .492 | 0.004 | 0.01 | -0.01 | 0.015 | .491 |
| 41-55 | 0.56 | 0.04 | 0.48 | 0.64 | <.001 | -0.04 | 0.005 | -0.05 | -0.03 | <.001 |
| 56-64 | 0.40 | 0.03 | 0.34 | 0.47 | <.001 | -0.05 | 0.005 | -0.06 | -0.04 | <.001 |
| **Female sex (ref = male sex)** | 1.15 | 0.06 | 1.05 | 1.27 | .003 | 0.009 | 0.003 | 0.003 | 0.01 | .004 |
| **Residence in zip code with lowest-quartile median income (ref = No)** | 0.83 | 0.06 | 0.73 | 0.95 | .006 | -0.01 | 0.004 | -0.02 | -0.003 | .005 |
| **Residence in zip code with concentrated poverty (ref = No)** | 0.97 | 0.11 | 0.78 | 1.21 | .812 | 0.00 | 0.01 | -0.01 | 0.011 | .81 |

**Abbreviations:** aOR, adjusted odds ratio; CI, confidence interval; LCI, lower confidence interval; UCI, upper confidence interval

**Supplementary Figure 6. Unadjusted Rates of Health Insurance Transition for Sensitivity Analysis Combining Traditional Medicaid and Medicaid Managed Care for Individuals with Schizophrenia by Health Insurance Type at Start of Period**

**Note:** N = 36,754 person-period observations. Standard errors are clustered at the 5-digit ZIP code level. *Indicates a statistically significant difference in predicted probability from the reference group (Marketplace insurance) at the 5% level. 95% Confidence intervals for each group are shown with vertical bars.

**Supplementary Figure 7. Health Insurance Transitions over the Study Period for Sensitivity Analysis Combining Traditional Medicaid and Medicaid Managed Care**

**Note:** For display purposes, all combinations with fewer than 11 observations were dropped (N = 353; 1.0%). Among those with Marketplace and Health Safety Net insurance at month 1, 6.7% and 6.3% were dropped, respectively.

**Supplementary Table 4. Full Regression Results for Sensitivity Analysis Excluding the 2014 to 2015 period from Analysis**

| **Outcome:** Any Transition | **aOR** | **Standard Error** | **95% CI** | | ***P* value** | **Average Marginal Effect** | **Standard Error** | **95% CI** | | ***P* value** |
| --- | --- | --- | --- | --- | --- | --- | --- | --- | --- | --- |
|  |  |  | **LCI** | **UCI** |  |  |  | **LCI** | **UCI** |  |
| **Health insurance type at start of period (ref = Marketplace)** |  |  |  |  |  |  |  |  |  |  |
| Private | 0.20 | 0.02 | 0.16 | 0.25 | <.001 | -0.25 | 0.02 | -0.30 | -0.21 | <.001 |
| Traditional Medicaid | 0.59 | 0.07 | 0.48 | 0.74 | <.001 | -0.11 | 0.02 | -0.15 | -0.06 | <.001 |
| Medicaid managed care | 0.13 | 0.01 | 0.10 | 0.16 | <.001 | -0.29 | 0.02 | -0.33 | -0.24 | <.001 |
| Health Safety Net | 0.86 | 0.11 | 0.67 | 1.11 | .251 | -0.03 | 0.03 | -0.09 | 0.02 | .253 |
| Medicare Advantage | 0.12 | 0.03 | 0.07 | 0.21 | <.001 | -0.29 | 0.03 | -0.34 | -0.24 | <.001 |
| Integrated Medicare & Medicaid | 0.33 | 0.07 | 0.23 | 0.49 | <.001 | -0.20 | 0.03 | -0.26 | -0.13 | <.001 |
| **Age, y (ref = 18-25)** |  |  |  |  |  |  |  |  |  |  |
| 26-40 | 0.99 | 0.06 | 0.89 | 1.11 | .893 | 0.00 | 0.01 | -0.02 | 0.01 | .894 |
| 41-55 | 0.63 | 0.04 | 0.56 | 0.71 | <.001 | -0.06 | 0.01 | -0.07 | -0.04 | <.001 |
| 56-64 | 0.50 | 0.03 | 0.44 | 0.57 | <.001 | -0.08 | 0.01 | -0.10 | -0.07 | <.001 |
| **Female sex (ref = male sex)** | 1.05 | 0.04 | 0.98 | 1.13 | .2 | 0.01 | 0.004 | -0.003 | 0.01 | .202 |
| **Residence in zip code with lowest-quartile median income (ref = No)** | 0.95 | 0.06 | 0.84 | 1.07 | .419 | -0.01 | 0.01 | -0.02 | 0.01 | .419 |
| **Residence in zip code with concentrated poverty (ref = No)** | 1.08 | 0.14 | 0.84 | 1.39 | .56 | 0.01 | 0.02 | -0.02 | 0.04 | .567 |

**Abbreviations:** aOR, adjusted odds ratio; CI, confidence interval; LCI, lower confidence interval; UCI, upper confidence interval

**Supplementary Figure 8. Adjusted Rates of Health Insurance Transition for Sensitivity Analysis for Individuals with Schizophrenia by Health Insurance Type at Start of Period Changing the Start of Period to April 1**


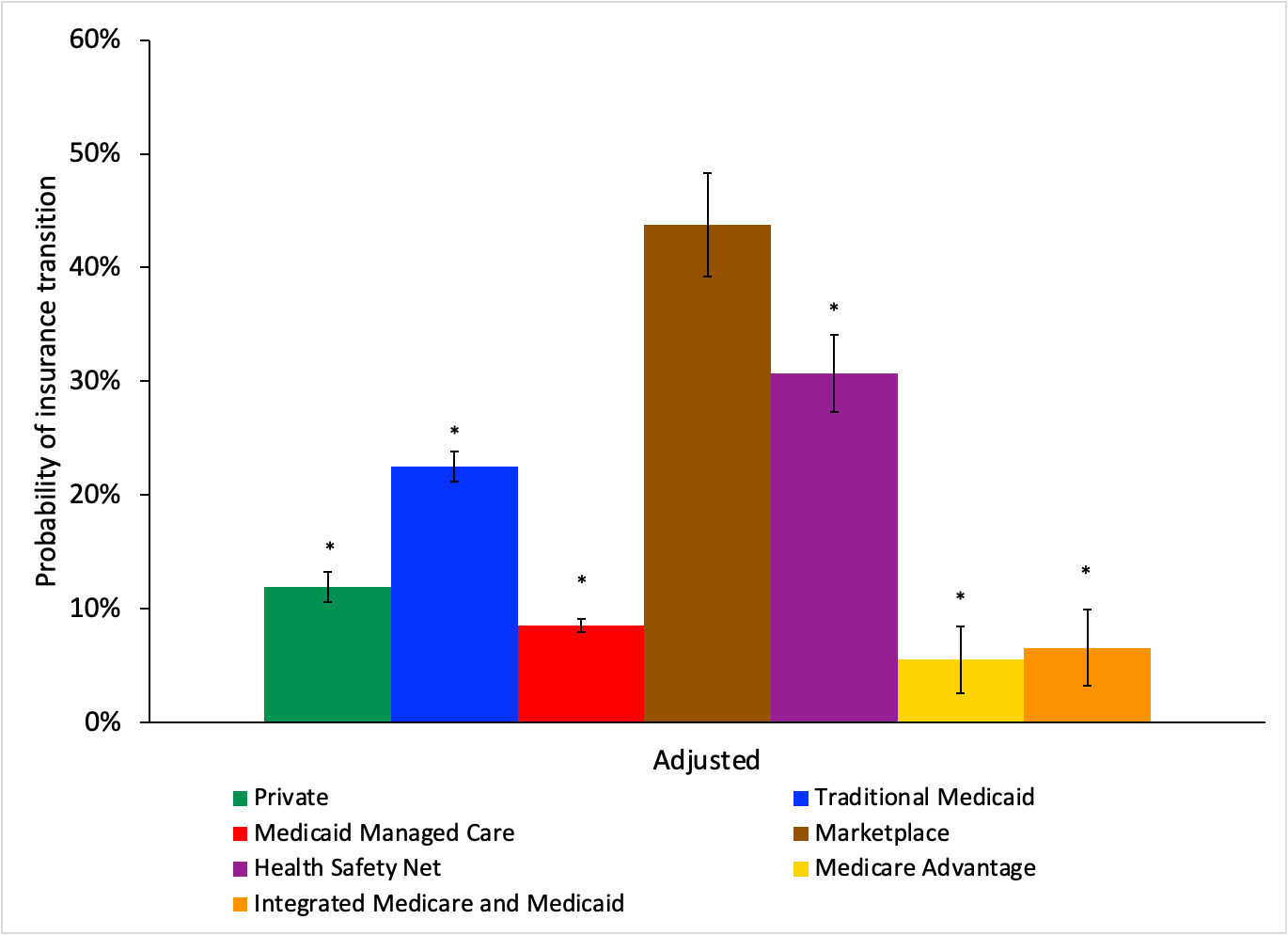


**Note:** N = 27,094 person-period observations. Adjusted results control for age, sex, residence in a ZIP code in the lowest quartile of median income, and residence in a ZIP code with concentrated poverty. Standard errors are clustered at the 5-digit ZIP code level. *Indicates a statistically significant difference in predicted probability from the reference group (Marketplace insurance) at the 5% level. 95% Confidence intervals for each group are shown with vertical bars.
